# 18 individual genes underwent variant screening in a northwest Chinese group comprised 83 probands diagnosed with early-onset high myopia

**DOI:** 10.1371/journal.pone.0329472

**Published:** 2025-09-08

**Authors:** Yang Liu, Shao-Chi Zhang, Wen Zhang, Zhong-Qi Xue, Yi-Xuan Qin, Shun-Yu Piao, Wen-Jing Li, Meng-Li Ji, Wen-Juan Zhuang

**Affiliations:** 1 People’s Hospital of Ningxia Hui Autonomous Region, Ningxia Institute of Clinical Medicine, Yinchuan, China; 2 People’s Hospital of Ningxia Hui Autonomous Region, Ningxia Eye Hospital, Yinchuan, China; 3 Department of Ophthalmology, Affiliated Hospital of Qingdao Binhai University, Qingdao, China; Hebrew University Hadassah Medical School, ISRAEL

## Abstract

**Purpose:**

To investigate the variants in 18 disease-causing genes associated with nonsyndromic myopia in 83 Chinese individuals diagnosed with early-onset high myopia(eo-HM).

**Methods:**

Variants in 18 candidate genes in 83 probands with eo-HM were distinguished by whole-exome sequencing (WES) and assessed by multistep bioinformatics analysis.

**Results:**

Four likely pathogenic variants were detected in 4 of the 83 probands (4.8%) with eo-HM. All of these are missense variants, such as (NM_014452: c.443C > T) in *TNFRSF21*, (NM_013291: c.799C > G) in *CPSF1*, (NM_201269.3: c.3266A > G) in *ZNF644*, and (NM_001135195: c.577G > A) in *SLC39A5*. These variants were verified by Sanger sequencing, and all allele frequencies were less than 0.01 in the 1000G, ExAC, ESP6500, and gnomAD databases. In addition, the pathogenicity of these variants was determined using several computational tools including SIFT, Mutation Taster, Polyphen-2, PROVEAN, M-CAP, CADD, and DANN. However, it should be noted that the Tyr1089Cys variant was classified as neutral solely using PROVEAN.

**Conclusion:**

Our findings support the hypothesis that the variants observed in *TNFRSF21, CPSF1, ZNF644,* and *SLC39A5* are the causative genes of eo-HM and expand the spectrum of eo-HM variants observed across various ethnic groups. The dissemination of knowledge on the impact of *TNFRSF21, CPSF1, ZNF644,* and *SLC39A5* on eo-HM is under investigation.

## Introduction

High myopia, which represents a serious type of myopia, is the main reason of severe ocular complications [1]. Currently, the condition of high myopia remains serious, and it is expected that there will be a significant elevation in the total count of people with high myopia from 399 million throughout the year 2020–516 million by 2030 [2]. The situation in East Asia is even worse. The occurrence of high myopia was 21.6% in Korea, 21% in the Singaporean [3–4].The proportion of high myopia in China also increased from 7.9% to 16.6% [5].

It is widely acknowledged that the onset of high myopia is influenced by genetic and environmental variables [6]. Early-onset high myopia (eo-HM), observed in children under the age of 7 [7], is thought to be influenced by minimal environmental factors, such as lifestyle, diet, and UV light exposure, which are suggested to contribute to the onset and progression of non-syndromic myopia [8]. Genetic factors are the determined factor in the development [9]. Compared with late-onset myopia (lo-HM), which usually occurs in children aged 7 and above [10], it is closely related to environmental factors such as prolonged near work, limited outdoor activities, and inadequate exposure to natural light, all of which have been shown to contribute to the onset and progression of myopia [11]. Therefore, Compared to other forms of myopia, early-onset high myopia (eo-HM) is more strongly associated with genetic factors, which have been identified through extensive research and are crucial in understanding the underlying genetic contributions to the disease.

Up to now, with the development of whole-exome sequencing (WES), it has been reported that 18 genes were potentially causative for non-syndromic high myopia, such as eleven genes related to autosomal dominant: *ZNF644* [12]*, SCO2* [13]*, SLC39A5* [14]*, CCDC111* [15]*, P4HA2* [16]*, BSG* [17]*, CPSF1* [18]*, NDUFAF7* [19]*, TNFRSF21* [20]*, XYLT* and *DZIP1* [21]; four genes related to recessive inheritance, *LRPAP1* [22]*, CTSH* [23], *LEPREL1* [24] and *LOXL3* [25]; and three X-linked genes, *ARR3* [26]*, ARR4* [27] and *OPN1LW* [28]. Combined with these previous investigations, we examined variants in the 18 known genes in a group of 83 northwest Chinese families suffering from eo-HM in order to enlarge the current genetic spectrum in different ethnic groups.

## Materials and methods

### Patient recruitment

Eighty-three patients from 83 unrelated families were admitted in this investigation from 21/5/2020–30/1/2024. All patients were diagnosed with eo-HM based on high myopia ≤ –6.00D after mydriasis, and the onset of high myopia presents less than 7 years old with no ocular or systemic disease [7]. Nuclear families collected have been obtained from the Ningxia eye hospital of Ningxia Hui Autonomous Area in the northwest of China, clinical information of 83 patients with high myopia have been show in S1 Table. After receiving written informed consent from each patient or their parents, genomic DNA was extracted from peripheral blood samples and the detailed clinical examinations were conducted. The present investigation followed the standards defined in the Declaration of Helsinki and has been approved from the institutional review board of People’s Hospital of Ningxia Hui Autonomous Region(2020-KY-GZR019).

### WES and analyses

Genomic DNA was extracted from peripheral blood samples using a standard phenol–chloroform method. Whole exome sequencing (WES) was performed using the SureSelect Human All Exon V5 Kit (Agilent Technologies, USA) according to the manufacturer’s instructions, to enrich coding exons and flanking intronic regions across approximately 50 Mb of the human genome. The captured DNA libraries were then sequenced on the Illumina HiSeq 2500 platform, generating 150 bp paired-end reads with a mean coverage depth of over 100 × , ensuring reliable variant detection [29].

Raw sequencing reads were processed using FastQC for quality control, and adapters and low-quality reads were removed using Trimmomatic. Clean reads were then aligned to the human reference genome (GRCh37/hg19) using BWA-MEM. Post-alignment processing, including duplicate marking, local realignment around InDels, and base quality score recalibration, was conducted using the Genome Analysis Toolkit (GATK) best practices workflow [30]. Specifically, GATK Table Recalibration was used to improve variant quality, and both single-nucleotide variants (SNVs) and insertions/deletions (InDels) were identified using the GATK Unified Genotyper.

To interpret functional consequences, variants were annotated using ANNOVAR, which classifies SNVs and InDels based on their genomic context and predicted effects on protein-coding sequences, as previously reported [31–32].

The filtration of WES data for the 18 genes from 83 probands in our study was performed along these lines: (1) alterations inside of the exonic and splicing site regions were extracted; (2) SNPs and indels (within 2 bp) with minor allele frequency (MAF) less than 0.01 within the 1000 Human Genome Project (1000G) (ftp://1000ge nomes.ebi.ac.uk/vol1/ftp), Exome Aggregation Consortium Database (ExAC) (http://exac.broadinstitute.org/), NHLBI Exome Sequencing Project (ESP6500) (http://evs.gs.washington.edu/EVS/) or the Genome Aggregation Database (gnomAD) (https://gnomad.broadinstitute.org/) were extracted; (3) synonymous alterations have no influence in splicing sites were eliminated; (4) missense variants anticipated to be benign utilizing various tools such as Variant Phenotyping v2 (Polyphen-2) (http://genetics.bwh.harvard.edu/pph2/), Sorting Intolerant From Tolerant (SIFT) (https://sift.bii.a-star.edu.sg/), Protein Variation Effect Analyzer (PROVEAN) (http://provean.jcvi.org/genome_submit_2.php), The MutationTaster2 (http://www.mutationtaster.org/) or Mendelian Clinically Applicable Pathogenicity (M-CAP) (http://bejerano.stanford.edu/mcap/) were eliminated; and (5) variants not heterozygous in Autosomal Dominant (AD) families, where the disease is typically inherited in a dominant pattern. Sanger sequencing validated the co-segregating conditions of the variants that remained after the filtering process (S1 Fig). Table 1 lists the primers designed for screening variants and validated using Sanger sequencing.

**Table 1:** PCR primers for sanger sequencing in this study.

primer name	Forward	Reverse
*DZIP1*-HM25	ACTCCCAGTGCTCGGTACAC	CTCTTTGCAAGAAGGTGCAG
*TNFRSF21*-HM26	GTGAGGTGGAGCTGGAGAAG	GGACCTTTACCAGGCATGAG
*CPSF1*-HM33	GGTACAACAGCGAGTTGACG	CCTCCTCATCCTGTTTGAGC
*OPN1LW*-HM129	GGAAATGCCCAGTGTCTGTT	GGACCACAGAGCCTTTCCTA
*ZNF644-*HM115	TTGTGGCTGATACATCAACGG	AACACACTTGTCAGCTCTGTGG
*SLC39A5*-HM95	TGAGCTCAGGCAATCTACCC	CTTCCAGGATTCAGGGTGTC

### Bioinformatics analysis

All the variants detected in this study were assessed using a comprehensive bioinformatics pipeline. Raw sequencing reads were aligned to the human reference genome (GRCh37/hg19) using BWA-MEM (v0.7.17) [33]. GATK (v3.8) was employed for base quality recalibration, indel realignment, and variant calling through the UnifiedGenotyper module [34]. Variant quality was evaluated using standard hard filtering parameters recommended by the GATK workflow.

Subsequently, variants were annotated using ANNOVAR, integrating multiple public databases including 1000 Genomes, ExAC, ESP6500 and gnomAD to determine population frequency, predicted pathogenicity, and clinical relevance. Functional effects were further interpreted using in silico tools such as SIFT, PolyPhen-2, MutationTaster and so on.

The selection of these tools and parameters was based on their performance in previous studies and compatibility with our data quality. SIFT [35]is used to predict the spatial conformational changes of proteins caused by gene variants based on gene sequence homology, which influences the protein’s role. Polyphenon [36] was used to predict the potential influence of amino acid substitutions on the composition and function of proteins. Mutation Taster [37] can evaluate the pathogenic possibility of changes in DNA sequence, it is not only used to predict amino acid changes, but also to predict the functional consequences of short insertion or deletion (indel) alterations or both, and variants across intron-exon boundaries. PROVEAN [38] tool was developed to predict the impact of protein sequence variants on protein function. M-CAP [39] can correctly eliminate 60% of rare and uncertain missense variants in typical genomes with 95% sensitivity. Combined Annotation Dependent Depletion (CADD) [40] (https://cadd.gs.washing ton.edu/snv) is a software utilized for the purpose of prediction, which combines the variant of alleles, the pathogenicity of variation and other factors to build a model to evaluate each variant site, and give a specific score, referred to as C-Scores. CADD creates an original scoring algorithm to measure the harmful degree of a variant site. The technique known as Domain-Adversarial Training of Neural Networks (DANN) [41](https://cbcl.ics.uci.edu/public_data/DANN/) uses a neural network and integration algorithm to predict damaging variants on the basis of SIFT, PolyPhen, PROVEAN, and Mutation Taster. To compare sequence conservation among the eight species, Clustal Omega [42] (https://www.eb i.ac.uk/Tools/msa/clustalo/) was employed. Furthermore, I-TASSER predicted the three-dimensional structure protein modeling of variants and wild-type proteins [43] (https://zhanglab.ccmb.med. umich.edu/) and Swiss-PDB Viewer visualized the protein structure.

## Results

After filtering variants across 18 candidate genes in a cohort of 83 individuals with eo-HM and multiple clinical features, six heterozygous variants were identified in *DZIP1, TNFRSF21, CPSF1, OPN1LW, ZNF644,* and *SLC39A5*, each found in six unrelated families.

**Fig 1:**
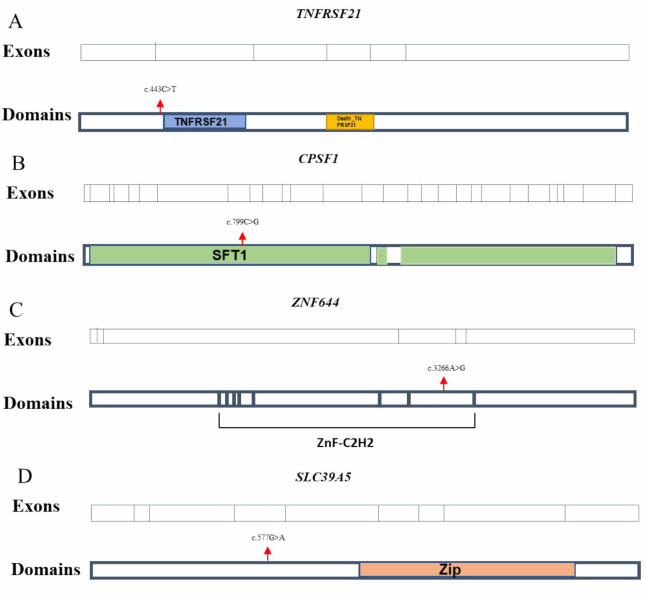
Location of the potentially pathogenic variants in *TNFRSF21, CPSF1, ZNF644* and *SLC39A5.* Exons of human *TNFRSF21, CPSF1, ZNF644* and *SLC39A5*(upper),and positions of variants corresponding to the protein model with functional domains highlighted(under).We identified four heterozygous variants in this study. The Gln267Glu in *CPSF1* were located in domain regions, but the other variants were not. TNFRSF21(colored blue) domain plays a role in T-helper cell activation, and may be involved in inflammation and immune regulation(A). SFT1(colored green) is involved in mRNA cleavage and polyadenylation specialization(B). C2H2 zinc fingers(colored purple) are the motifs take part in mitochondrial complex I activity **(C)**. The ZIP(colored orange) domain is contributed to the zinc transport **(D)**.

To validate these findings, Sanger sequencing was performed for all candidate variants in available family members. Notably, the Thr658Ala variant in *DZIP1* and the Leu153Met variant in *OPN1LW* were subsequently excluded from further consideration due to lack of co-segregation with the phenotype in the respective families, suggesting these variants are less likely to be pathogenic. The remaining four variants were further evaluated for pathogenicity based on the American College of Medical Genetics and Genomics (ACMG) guidelines [44], taking into account population frequency, computational predictions, segregation data, and functional domain involvement. These findings are discussed in the context of their potential roles in the genetic etiology of non-syndromic high myopia. There were four missense variants included *(*NM_014452: c.443C > T) in *TNFRSF21*, (NM_013291: c.799C > G) in *CPSF1*, (NM_201269.3: c.3266A > G) in *ZNF644* and (NM_001135195: c.577G > A) in *SLC39A5* for likely pathogenic (Table 2). Moreover, it should be noted that the Gln267Glu variant in *CPSF1* was positioned within domain locations, whereas the remaining variants were not situated in such locations (Fig 1). Furthermore, the scores of CADD and DANN were calculated in *TNFRSF21, CPSF1, ZNF644* and *SLC39A5* as 24.8,0.998; 23.2,0.993; 24.5,0.998 and 31,0.999, respectively.

**Table 2:** Summary of mutations in *ZNF644, SLC39A5, CPSF1, TNFRSF21,OPN1LW*and *DZIP1.*

Patients ID	Gene	Inheritance	Sex	Age at oneset	Age at exam	Refraction		Al		Chr.position	Exon	Mutation	Status	SIFT	PolyPhen2	PROVEAN	Mutation Taster	M-CAP	CADD score	DANN score	1000G		ExAC		ESP6500		genomAD	
						OD	OS												OD	OS	ALL	EA	ALL	EA	ALL	EA	ALL	EA
26	*TNFR SF21*	AD	F	EC	45	−21.00	−21.50	31.21	31.58	Chr6.47253985	2	c.443C > T p.T148M	Het	D	D	D	D	NA	24.8	0.998	None	None	0.001	0.0001	0.0005	0.0007	0.0015	None
33	*CPSF1*	AD	M	3	11	−6.00	−5.75	26.11	26.16	Chr8.145625775	8	c.799C > G p.Q267E	Het	NA	P	NA	D	NA	23.2	0.993	0.0014	0.002	0.0027	0.006	0.0005	None	0.0034	0.0055
115	*ZNF644*	AD	F	2	22	−9.75	−9.75	28.39	28.55	Chr1.91403464	4	c.3266A > G p.Y1089C	Het	D	D	N	D	D	24.5	0.998	0.0014	0.007	0.0003	0.0032	None	None	0.0002	0.0031
95	*SLC 39A5*	AD	M	EC	21	−7.25	−6.75	26.75	26.45	Chr12.56628713	4	c.577G > A p.D193N	Het	D	D	D	D	D	31	0.999	0.0002	0.001	0.00007462	0.0003	None	None	0.00007319	0.0003

F, female; Het, heterozygous; M, male; N, neutral; OD, right eye; OS, left eye; Chr, Chromosome; D,damaging; EA,East Asia;EC,early childhood; NA, not applicable; P, probably damaging.

### *TNFRSF21* variant

**Fig 2:**
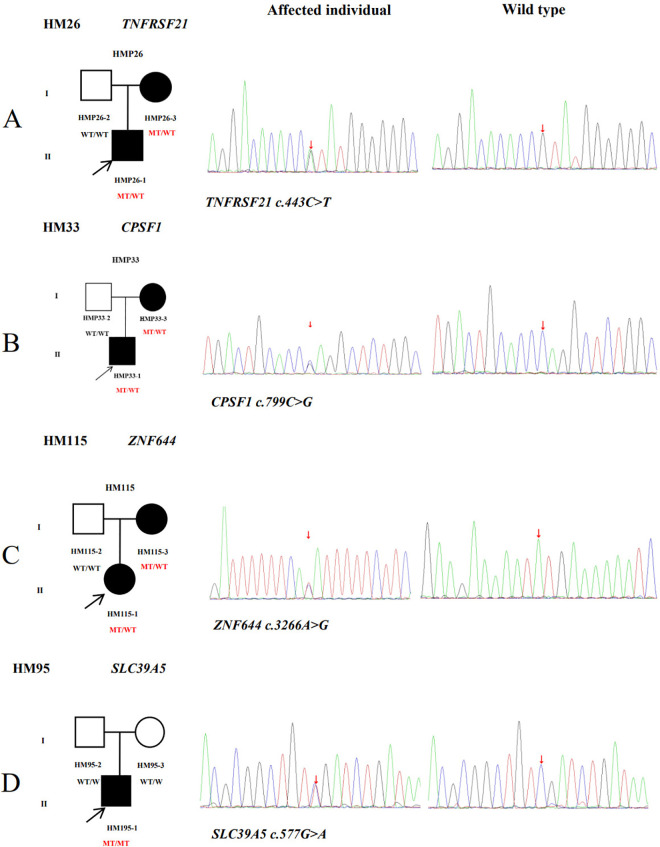
Four potentially pathogenic variants detected in this study. The black arrow represents the patient. WT: Wild type; MT: Mutation. From left to right: pedigree plots of variants, sequences from affected individual with identified variant, sequences from wild type, clinical information regarding the segregation of the identified variants within the families is included in S1 Table.

**Fig 3:**
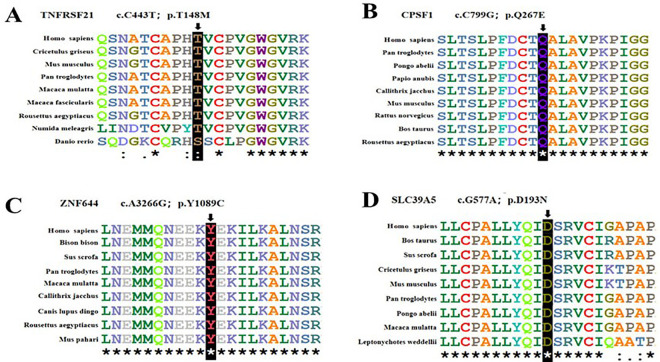
Conservation analysis revealed evolutionary conservation of the variant. It shows that multiple alignments of the amino acids from different species. The arrow indicates the location of the variants(A-D).

**Fig 4:**
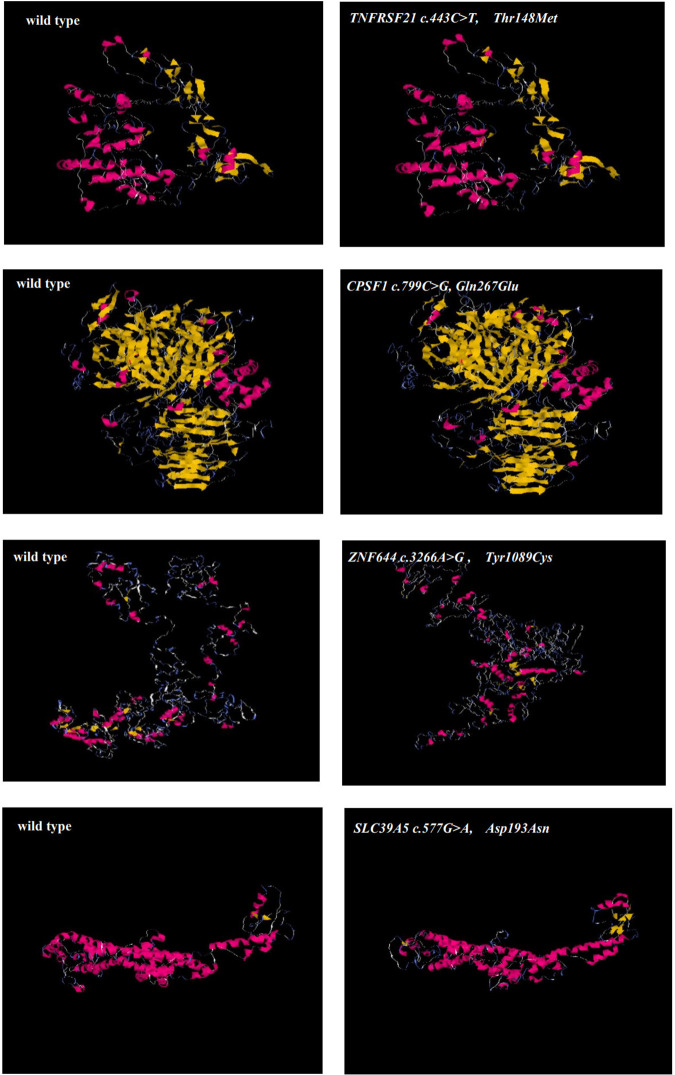
Predicted three-dimensional structure of proteins. Predicted crystal structures of wild type(left) and variant (right)proteins. Yellow shows residue of wild type and variant, green represents residues interact with wild type(left) and variant residue(right)(A-D).

One heterozygous variant in *TNFRSF21* were found from family26 (Fig 2A). Before the age of seven, the patient and affected family member had high myopia. During the examination, it was observed that the right eye had a high myopia measuring −21.00 D, accompanied by an ocular AL of 31.21 mm. Similarly, the left eye exhibited a myopia measuring −21.50 D, accompanied by an ocular AL of 31.58 mm. The missense variant identified in *TNFRSF21* (NM_014452: c.443C > T) was evaluated using various computational tools, including SIFT, Polyphen-2, PROVEAN, MutationTaster2, and M-CAP. Collectively, the results of these analyses suggest that this variant is likely to have a damaging effect. Neither was detected in the 1000 Genomes (1000G) or gnomAD-east databases. The substitution Thr148Met showed high conservation when compared across various sequence alignments in species that were homologous (Fig 3A). This finding highlights the significant role of this site in protein functions. Interestingly, the three-dimensional structure of the protein did not show any obvious changes in its function due to the identified mutation(Fig 4A).

### *CPSF1* variant

A missense variant was detected in *CPSF1* in an adolescent aged 11 years (Fig 2B) with low vision in childhood according to his parent’s narration. The diopter of both eyes is close to −6.00 D and the axial length of both eyes is more than 26 mmm without oculopathy or systemic disease. A novel missense variant c.799C > G was detected in exon 8 and was suggested to be a likely pathogenic symptom via Polyphen-2, SIFT, PROVEAN, and MutationTaster2. The variant is located on the SFT1 superfamily mRNA cleavage and polyadenylation specificity factor, which may play a role in RNA processing and modification. In addition, Gln267Glu showed highly conserved amino acid residues in various species (Fig 3B) and new hydrogen bond formation in the protein function from its three-dimensional structure (Fig 4B). This alteration is very rare, with a population frequency of 0.01% according to gnomAD. The frequencies of this alteration in other databases are also presented in Table 2.

### *ZNF644* and *SLC39A5* variants

(NM_201269.3: c.3266A > G) within *ZNF644* and (NM_001135195: c.577G > A) within *SLC39A5* have been detected through family 115 and 95, which were verified by family co-segregation (Fig 2C, D). These variants occurred at an extremely conserved region in eight species (Fig 3C, D). According to SIFT, Polyphen-2, MutationTaster2, PROVEAN and M-CAP, the c.3266A > G in *ZNF644* and c.577G > A in *SLC39A5* were disease-causing, except the c.3266A > G in *ZNF644* was neutral by PROVEAN. Both of these variants were detected as rare SNPs in ExAC. The substitutions are predicted to have a significant impact on the protein fold and stability in the three-dimensional structures of *ZNF644* and *SLC39A5* (Fig 4C, D).

## Discussion

To date, at least 18 causative genes associated with high myopia have been discovered [12–21], however, no related studies have been conducted in populations from Northwest China. In this study, we screened 18 causative genes in 83 patients from 83 unrelated families using WES, four likely pathogenic variants in *TNFRSF21, CPSF1, ZNF644* and *SLC39A5* were detected. All alterations that occurred within these genes affected the coding region role and were not found or rare in four observed databases (ExAC,1000G, ESP6500 and gnomAD). PolyPhen-2, SIFT, PROVAN, Mutation Taster, and M-CAP assessed the impact of amino acid changed by sequence variant, respectively. CADD combines multiple factors such as allelic variant and variant pathogenicity to evaluate each variant site while DANN uses neural network algorithms to evaluate the harmfulness of variants.

Pan [45] *etal* first discovered *TNFRSF21* is associated with high myopia through a large Chinese family, a novel missense variant Pro146Ala was identified and three uncommon heterozygous alteratioms (Pro202Leu, Glu240Ter and Ala440Gly) in *TNFRSF21* were observed in the screening of 220 unrelated individuals with HM in the same study. The Pro146Ala variant was found to significantly enhance the proliferation of adult retinal pigment epithelial cell line-19 cells in comparison with the wild type. Herein, we found a heterozygous missense variant Thr148Met in *TNFRSF21*, since the Pro146Ala variant might result in HM via the regulation of the apoptosis of myopia-related cells [46], the distance between Thr148Met and Pro146Ala is very close, whether Thr148Met acts on myopia in the same mechanism as Pro146Ala needs further explored.

A total of 6 variants (Phe1291Ter, Val943LeufsTer65, Gln620Ter, Tyr5Ter, Asp1275Tyr and c.4146-2A > G) within CPSF1 have been recognized in 6 of 623 probands suffering from eo-HM and two alterations were proved to be related to retinal ganglion cell in zebrafish in previous study [47]. Herein, we found a novel missense variant Gln267Glu in *CPSF1.* which located in the SFT1 domain, It is postulated that this substitution could potentially impact the stability of the protein construction. Further studies of how the variant of *CPSF1* is connected with high myopia are still needed.

*ZNF644*, which is positioned on chromosome 1p22.2 and includes 6 exons, functions as a transcription factor with C2H2 Kruppel type zinc finger domains [48]. This protein underwent expression not only in the human retina and the pigment epithelium of retina, but also appears to have a function in ocular wall development. Elongation of the eye’s axial length was revealed to be a hallmark characteristic of high myopia [49]. The change of *ZNF644* proteins may have an effect on normal eye development and contribute to the axial elongation seen in high myopes since it is hypothesized that *ZNF644* consider to be a transcription factor that controls genes expression which implicated in the process of ocular development [50]. In a study by Shi et al. [12], a missense variant in *ZNF644* (Ser672Gly) was first identified by WES throughout a five-generation Han Chinese family suffering from high myopia. In recent years, additional variants in *ZNF644* linked to high myopia have been reported in both China and America [51]. Herein, we distinguished a novel missense variant, Tyr1089Cys, in exon 4 of the *ZNF644* gene. Nevertheless, the specific mechanism underlying the action of *ZNF644* and its function in the pathogenesis of high myopia remains unclear. More functional investigations are required to be conducted.

Located at 12q13.3, SLC39A5 encodes a zinc transporter that belongs to the ZIP family known as solute carrier family 39 member 5. Its primary role is to maintain zinc homeostasis. [52]. In a study by Guo et al. [14], the initial discovery involved the detection of a truncation alteration within the *SLC39A5* gene (Tyr47Ter) that exhibited heterozygosity, and one proposal was made indicating that the (c.141C > G, Tyr47Ter) alteration may result in the impairment of *SLC39A5* features by generating a truncated protein that significantly boosts the expression levels of Smad1 at mRNA and protein. Smad1 is a vital transcription factor located downstream within the BMP/TGF-b transduction pathway. Several investigations have suggested a correlation between myopia and the BMP/TGF-b pathway, suggesting that interference with this pathway could cause refractive errors or potentially high myopia [53,54]. Additionally, *SLC39A5* was detected in each stage of the ocular development and exhibited an elevated expression levels in both the sclera and retina [14]. Employing a screening of screening 298 families suffering from early-onset high myopia, Jiang et al [23] distinguished yet another missense alteration (c.1238G > C, Gly413Ala). In addition, Feng et al [55] stated three other heterozygous missense variants in isolated cases (Arg84Trp, Pro287Leu, and Arg319Thr). Herein, we distinguished a novel variant in *SLC39A5*, The Asp193Asn variant in exon 4, located near the terminal exon, may lead to alterations in protein expression due to its effect on mRNA splicing or stability. Our findings further expand the variants spectrum of *SLC39A5* in early-onset high myopia.

Although four likely pathogenic variants were identified in this study, they account for only approximately 5% of the total cohort, indicating a relatively low diagnostic yield.

indicating a relatively low diagnostic yield. We further analyzed the possible reasons for this low diagnostic yield and provided insights for future research directions.

First, the sample size may be a key factor affecting the diagnostic yield. The small sample size may have limited our ability to represent all potential pathogenic variants. For larger sample sizes are generally necessary to increase the probability of identifying additional pathogenic variants [56]. Therefore, the sample size limitation may have impacted the range of genetic variants we were able to detect in this study.

Second, the methodology used, whole exome sequencing (WES), while effective in detecting variants in protein-coding regions, has inherent limitations. WES primarily focuses on exonic regions of the genome, meaning that non-coding regions, structural variants, and deep intronic variants may not be detected. Since these types of variants may play significant roles in the genetic basis of certain diseases [57], the limitations of WES could be a contributing factor to the low diagnostic yield.

Additionally, the complexity and heterogeneity of the disease itself may also influence the diagnostic yield [58]. In some genetic diseases, there may be considerable phenotypic diversity, and the genetic background may exhibit high heterogeneity, with pathogenic variants spread across multiple genes or involving rare and previously unreported variants. Even with WES, it may not be possible to comprehensively identify all pathogenic variants. Furthermore, disease development may be influenced by environmental factors, epigenetic modifications, and gene-environment interactions, which cannot be fully captured by current genetic screening methods [59], further complicating the diagnostic process.

Lastly, the low diagnostic yield may suggest that we have not yet fully understood the genetic basis of the eo-HM, indicating the possibility of undiscovered pathogenic variants. Nonetheless, this study provides valuable insights into the genetic mechanisms of eo-HM and lays the foundation for future research. Future studies can explore larger sample sizes, improve sequencing technologies, and delve deeper into non-coding regions and structural variants to uncover additional pathogenic variants. Although the diagnostic yield in this study was lower than expected, the results reflect the limitations of current technologies and our incomplete understanding of the genetic basis of eo-HM. This highlights the need for further research in genetic studies, emphasizing the importance of expanding genetic testing technologies and increasing sample sizes to improve diagnostic rates and gain a more comprehensive understanding of eo-HM’s genetic landscape.

The limitations of our study include:(1)Whole genome analysis can identify variants throughout the genome, including intronic and exonic regions. Although the exome accounts for only 1% of the genome, it has been estimated that a significant proportion of known disease-causing variants—especially those in Mendelian disorders—are located within coding regions. Therefore, WES remains a widely used and efficient tool for variant discovery in such contexts [60]. (2) Although we have used seven prediction software based on different principles to enhance the prediction accuracy of the pathogenicity of variants, these existing databases may be inaccurate or incomplete, and eventually affect the accuracy of pathogenicity prediction.

## Conclusion

Overall, by using of WES and bioinformatics, we identified 4 genetic variants associated with eo-HM development. To the best of our understanding, this is the initial investigation to screen the known high myopia gene variants in northwest Chinese. The results revealed 4 likely pathogenic variants related to eo-HM, offering further evidence of *TNFRSF21, CPSF1, ZNF644* and *SLC39A5* contributed to the eo-HM. Furthermore, our results have contributed to the broadening of the variant spectrum of eo-HM across various countries, thereby offering valuable insights for future genetic investigations pertaining to HM.

## Supporting information

S1 Table Clinical information of 83 patients with high myopia. (XLSX)
